# What Happens after Therapy? Quality of Life and Neurocognitive Functions of Children with Malignant Posterior Fossa Tumors after Adjuvant Therapy

**DOI:** 10.4103/0028-3886.329599

**Published:** 2021-09-01

**Authors:** Ujwal Yeole, Shantala Hegde, Mohit Gothwal, AR Prabhuraj, Sampath Somanna, K Thennarasu, Arivazhagan Arimappamagan

**Affiliations:** 1 Departments of Neurosurgery, Clinical Psychology, NIMHANS, Bengaluru, Karnataka, India; 2 Departments of Neurosurgery, Biostatistics, NIMHANS, Bengaluru, Karnataka, India

**Keywords:** Cognitive function, children, ependymoma, IQ, medulloblastoma, neuropsychology, posterior fossa tumor, quality of life

## Abstract

**Purpose:**

The health-related quality of life (HRQoL) is an important endpoint in modern clinical practice with improved survival of pediatric posterior fossa malignant brain tumors (PFMBTs). We evaluated the effect of environmental and psychosocial milieu on QoL and cognitive functioning (CF) of Indian children with PFMBT.

**Methods:**

In a cross-sectional study, 47 children <18 years of age with medulloblastoma or anaplastic ependymoma were evaluated ≥6 months after completion of adjuvant therapy. All clinical and socioeconomic details, educational status of child and family members, socioeconomic status, environmental factors affecting QoL were documented. Children underwent HRQoL evaluation using Pediatric quality of life Inventory (PedsQL) questionnaire and neuropsychological evaluation.

**Results:**

The median age of the cohort at presentation was 7 years (1−18) and median duration of evaluation after adjuvant therapy was 16 months. In 47 families, 72.34% had low monthly income and 76.6% of mothers took formal education. QoL scores were above median values. Parents reported scores highlighted that Lansky performance score (*P* = 0.001) and maternal education (*P* = 0.043) significantly influenced the cognitive component of QoL. Twenty-seven children had below-average IQ. Young age at presentation (*P* = 0.020), maternal education (*P* = 0.032), high socioeconomic status (*P* = 0.001) influenced the IQ score. Even though the majority of children (57.44%) had below-average IQ, they had a score of more than 50 on the cognitive functioning scale. A total of 72.5% of the eligible children in our cohort went back to school following therapy, though often with a delay of one academic year.

**Conclusions:**

Overall cognitive functioning scores of these children are good, but they are not representative of actual neurocognitive tasks based performance or IQ scores. Children should remain under regular follow-up with a neurocognitive assessment and psychological counseling at regular intervals.

Nervous system tumors are the second most common childhood tumors after leukemia.^[1]^After astrocytomas (25.1%), medulloblastomas (20.6%) and ependymoma (9%) are the commonest posterior fossa tumor affecting children in the first decade of life.^[2]^ With the improvement in treatment modalities, the survival of malignant posterior fossa brain tumors (PFBTs) has significantly increased in the last few decades.

Much has been researched about health-related quality of life (HRQoL) of adults with cancer.^[3]^They have unique symptoms and problems and HRQoL is an important endpoint in modern-day clinical practice^[4]^ However, similar work in pediatric brain tumors is sparse, probably due to conceptual and methodological issues. Cognitive deficits due to the tumor, surgical procedure, and adjuvant therapy posea major challenge in evaluating these children. There are only a handful ofstudies carried out mainly in the Western population which tried addressing these issues and to validate reliable methods of assessment of QoL and neurocognitive effects. They also attempted formulating protocols to deal with these problems in terms of regular follow-up and assessment for neurocognitive functioning, special school education, cognitive support groups for children and their parents^[5,6]^

In the Indian scenario, following completion of adjuvant therapy, the overall outcome in terms of QoL, getting back to school, and daily life of social activities are not well documented. A multitude of factors can be responsible for QoL, which may vary due to geographical, social, economic, and cultural influences. Perhaps, extrapolating the findings from the Western studies would not provide the true picture owing to differences in socio-cultural and economic factors contributing to the overall QoL. This study was therefore carried out to examine the effect of environmental and psychosocial milieu on QoL and cognitive functioning in children with malignant posterior fossa tumors in our country following the completion of adjuvant therapy.

## Methods

Children up to 18 years of age at presentation, who had undergone treatment for histopathologically proven malignant PFBT from the year 2010 onwards at our institute and those who had completed a minimum of 6 months following adjuvant therapy were included in the study. The pre-operative and post-operative clinical details, present educational and occupational status along with socioeconomic background and educational and occupational details of family members, affecting QoL of the child were documented. The study protocol was approved by the Institute Ethics Committee (NIMH/DO/BS and NS 1^st^ meeting/2016, dt. 25.11.2016). Informed consent for participation in the study was obtained from the parents. This was a cross-sectional study, in which these patients underwent a one-time assessment of QoL and cognitive functions prospectively.

All patients underwent HR-QoL and cognitive function evaluation using the Pediatric quality of life Inventory (PedsQL) questionnaire from MAPI research trust, Lyon, France by James W Varni after obtaining due permission. (PedsQL™, Copyright © 1998 JW Varni, Ph.D. All rights reserved.) This questionnaire was selected as it provides a set of questions assessing specific domains in QoL. The brain tumor module^[7]^ of the Peds QL was used, which constitutes cognitive problems, pain and hurt, movement and balance, procedural anxiety, nausea, worry and cognitive functioning in cognitive functioning scale.^[8–10]^ The questionnaire is pertinent to different age groups namely 2−4, 5−7, 8−12, and 13−18 years. It uses both child and parent-reported outcome and they are either interviewer-administered for younger children or self-administered for older children and parents.The administration of the survey was performed in the outpatient department within 20 to 30 minutes of time.

An expert clinical neuropsychologist performed neurocognitive evaluation in a one-on-one session with the child in the presence of parents. Tests were administered based on the amenability for a detailed performance-based neurocognitive evaluation, such as the Binet-Kamat Intelligence Test (BKT)^[11]^/Raven’s Color Progressive Matrices (CPM)^[12]^/Raven’s Standard Progressive Matrices (SPM)^[13]^/Weschler’s Intelligence Scale for Children-Fourth Edition (WISC-IV Indian adaptation).^[14]^ If the child was not amenable, then evaluationwas carried out with a detailed interview of the parents/primary caregivers regarding the child’s overall socioadaptive functioning using the Vineland Social Maturity Scale (VSMS).^[15]^ Data analysis was done using SPSS software version 24. The significance of parameters was considered when the ‘p’ value was less than 0.05.

## Results

### Demography and clinical parameters

The study was carried out on children operated at our institute from January 2010 to December 2015. During this period, 178 cases of malignant PFBT had been operated at our institute. Sixty-four patients could be contacted for the study; the rest had either been not reachable or had expired. Of 64 patients, 17 patients did not come for follow-up and evaluation for the study. Forty-seven patients came for follow-up during the assessment period (2017) and consented for the evaluation. Therefore, this prospective cross-sectional study included 47 children with either medulloblastoma or anaplastic ependymoma, comprising of 31 (66%) male children. The mean age was 8.2 years (range: 1−18 years). On imaging, 38 (80.9%) tumors were located in the midline with 32 (68.1%) in vermis and 6 (12.8%) in the brain stem. Thirty-nine (83%) children underwent pre-operative ventriculoperitoneal (VP) shunt for hydrocephalus. Following surgery, 14 (29.8%) patients had a residual tumor on the immediate post-operative scan. Thirty-five (74.46%) had no fresh deficits postoperatively. Mutism was seen in 4 (8.51%) children[Table 1].

The Lansky performance score (LPS) for children was scored at presentation, discharge, and follow-up. At presentation, most had a LPS of 70 (n = 33; 70.2%). At discharge, none had a score of 50 or less and the majority of the children had score of 70 (n = 13; 27.7%) or 80 (n = 26; 55.3%).

All the patients except one had received radiation therapy following surgery. Thirty-one (66%) patients received chemotherapy. During follow-up, 9 (19.1%) cases had a recurrence, out of which, 4 (8.5%) had symptomatic and 5 (10.6%) had asymptomatic recurrence diagnosed on follow-up imaging. One patient with symptomatic recurrence had multiple intracranial and spinal diffuse metastasis.

### Educational and socioeconomic status

The return to school after therapy was assessed. Eighteen children had not joined the school after therapy. Out of these, seven were still less than five years at follow-up, and were not eligible to join. Out of the remaining 11, eight children were evaluated within 12 months from completion of adjuvant therapy, one had severe ataxia with bilateral cerebellar signs and others had very poor vision, bilaterally. One boy never joined school although he was seven years old at follow-up. A total of 29 (61.70%) children had gone to school following the completion of treatment.

To assess the impact of parental education on children’s cognitive outcomes, formal years of schooling was enquired for family members. Any level of school education was considered a formal education. Thirty-five children had both parents educated, while in the remaining 12 at least one parent was educated. Thirty-six (76.59%) mothers were educated. Out of 47 families, 34 (72.34%) were below poverty Line (BPL) earning less than 20,000 rupees per year.

### Quality of life assessment and the impact of clinical factors

The mean age at evaluation was 10.89 years (median age: 11 ± 4.91 years; range 3−21 years).The mean time period from completion of adjuvant therapy to the time of evaluation was 31.06 months (Median: 16 months).Impact of various clinical factors like tumor location, LPS at discharge, chemotherapy, recurrence, duration post-adjuvant therapy, shunt, socioeconomic status, maternal education were analyzed statistically against domains of QoL.

I] Cognitive Problem: Statistically, none of the clinical parameters had a significant impact on cognition as per child report. Higher maternal education and LPS of >70 at discharge were associated with lesser cognitive problems in children, though not reaching statistical significance. Parents-reported scores highlighted the findings that LPS (*P* = 0.001) and maternal education (*P* = 0.043) significantly influenced the cognitive component of QoL. Above poverty line (APL) families had lesser cognitive problems in their children, though not reaching statistical significance [Tables 2 and 3].

II] Movement and Balance: As per child and parent-reported scores, recurrence (*P* = 0.036 and *P* = 0.007, respectively) was the only clinical parameter affecting movement and balance. Also, children assessed at a longer duration (>12 months) post therapy had lesser problems as expected with better physical recovery. Children from APL families (*P* = 0.070) also had a lesser problem with ambulation probably due to the active part played by parents in physical rehabilitation and understanding of supportive treatment modalities like physiotherapy. [Tables 2 and 4].

III] Worry: Child-reported worry in relation to recurrence and usefulness of treatment was less in families with higher maternal education (*P* = 0.032) and families with a better socioeconomic background (APL). Parents of children with better Lansky score at discharge (*P* = 0.002) had less worry about the disease recurrence and outcomes of treatment. [Tables 2 and 5].

IV] Cognitive Functioning: Overall scores across all age groups for cognitive functioning was above the median. Clinical parameters did not have any significant impact on cognitive functioning as per children reports.

As per parent reports, children with better LPS (*P* < 0.001) had better cognitive functioning as probably they could get back to school and do better in their school work due to good physical condition. Higher maternal education (*P* = 0.087) leads to better environmental stimulation for improved cognition [Tables 2 and 6].

### Neurocognitive evaluation

The neurocognitive evaluation was aimed to assess the current level of intelligence or equivalent socioadaptive functioning quotient following completion of the treatment, which was then compared with other outcome measures such as quality of life scores. An IQ score <90 is considered as below average and an IQ score >109 is considered as above average. Twenty children had average or above-average intelligence while the remaining 27 were below average intelligence. This was further sub grouped based on Weschler’s Intelligence scale.

### Comparative analysis with clinical parameters

In our study, 27 children had below-average IQ, out of which 14 were boys. In 20 children with average or above-average intelligence, 17 were boys (*P* = 0.029). The mean age of children with average or above-average IQ was 6.5 years (range: 2−-9.5 years) while those children with below-average IQ had a mean age of 9.48 years (range: 5−13 years) (*P* = 0.020).

Twenty-seven children had been assessed more than 12 months after treatment completion. Among these children, 18 (66.7%) had below-average IQ, while the remaining 9 (33.3%) children had average or above-average IQ. Out of the 27 children with below-average IQ, 21 (77.8%) children had a mother with less or no education (*P* = 0.032).This is likely indicative of the importance of environmental stimulation provided in a household by educated mothers which helps children to be more active intellectually. In 13 APL families in the study, 11 children had average or above-average IQ (*P* = 0.001). This statistical significance is probably due to a better familial environment and also better education facilities available for these families.

The overall score of cognitive functioning for all children, as evaluated by PedSQL, was good, both as per child and parent reports. Children with below-average IQ had lesser scores (child-reported scores) than average or above-average IQ children.(*P* = 0.042). Even though the majority of children (57.44%) had below-average IQ by neuropsychological assessment, they had a score of more than 50 on the cognitive functioning scale. This implies good functioning of these children for daily life activities, but when specific cognitive tasks are assessed they fare poorly compared to children with average or above-average IQ.

## Discussion

As the survival in children with a brain tumor is improving, the emphasis has been slowly but steadily shifting towards the achievement of a better quality of life, than just mere survival. We studied the quality of life and current levels of neurocognitive functions of the children following adjuvant therapy for posterior fossa malignant brain tumors in our setting. Various factors that possibly influence the QoL and cognitive status were evaluated.

Our prospective study included 47 patients, which are a subset of patients treated during the selected study period. As our institute is a tertiary neurosurgical center, many parents prefer to undertake adjuvant therapy and follow up at their local facilities, making prospective evaluation at long term follow up period very challenging. The study results may not reflect the actual situation in view of possible selection bias, however, it does portray the pattern of QoL and cognitive functions of children at follow up and provides an opportunity to intervene and improve the social functioning of these children, based on the results. Some studies have evaluated the impact of age at diagnosis on QoL. Kulkarni *et al*. reported no significant impact of age at diagnosis in PFBT on QoL (*P* = 0.86),^[16]^ Bhat *et al*. reported HRQOL of children with no impact of age at diagnosis on QoL.^[17]^ We also noted that the age at presentation did not affect QoL scores, suggesting that the children were able to function without much problem in their daily life. Although parent-reported scores are less compared to child-reported scores, it is not significant. Correlating the IQ of the children with age at treatment, a critical review, which included 10 studies, noted that 8 studies showed lower levels of IQ when treatment was started at a younger age, though criteria of the young age were different ranging from 3−8 years. To the extent reviewed, four studies hitherto have shown statistical significance while 4 others were purely descriptive in nature.^[18]^

Ellenberg *et al*. demonstrated that tumor location impacting IQ scores was significant in hemispheric tumors and not in posterior fossa tumors.^[19]^ Bhat *et al*. found that tumor location was not significantly associated with total HRQoL.^[17]^ Kulkarni reported a significant impact of HCP on QoL of children (*P* = 0.03). QoL was substantially better in children in children without HCP.^[16]^ We noted that the tumor location or presence of hydrocephalus at initial evaluation for tumor surgery did not affect QoL and IQ levels. In our study, 38 (80.9%) tumors were located in the midline. Tumor location did not have any significant impact on QoL and IQ levels (*P* = 0.579). An ideal evaluation of QoL and cognition preoperatively and later following treatment of hydrocephalus and following adjuvant therapy would have enabled the accurate influence of hydrocephalus on QoL and cognition. It would also help if the QoL/IQ could be assessed in patients who develop shunt malfunction in the follow-up period. Our study indicated that we did not notice any significant difference in QoL and cognition functions long term between those who had hydrocephalus at presentation and those who did not. This implied that hydrocephalus did not have a long-term effect on patients, once it is taken care of by CSF diversion.

In our study, children with better LPS had lesser cognitive problems and lesser worry as per parent-reported scores. At the same time, the child-reported scores did not show any significance. On the contrary, neurocognitive scores (IQ/SQ scores) of children was not influenced by Lansky performance scores.

Probably a better performance score gives children the ability to engage in more interactive and social activities, which leads to better environmental cognitive stimulation. Fewer worries by these parents about their children, allow them to encourage children for psychosocial activities, and engage in school performance-based tasks. As IQ is not affected by performance scores, even children with poor performance in terms of physical activities can do better in terms of intellectual functioning.

The effect of adjuvant therapy on cognitive functions and QoL can be evaluated best by pre and post-therapy assessment, in a longitudinal model. In our cross-sectional study, almost all the children have received RT, and 66% of children received chemotherapy. Chemotherapy is the only treatment modality that affects nausea (*P* = 0.067). IQ scores were not influenced by chemotherapy (*P* = 1.000).The QoL dimensions of children were not significantly impaired compared to normative data, despite having undergone adjuvant therapy, though it may be difficult to ascertain this in the absence of pre-treatment evaluation. We planned the study in such a way that QoL and cognitive function evaluations were performed at least 6 months after the completion of RT/chemotherapy so that the acute effects of therapy do not confound the results. In various studies previously it has been substantiated that, RT, especially at young age, leads to a decrease in IQ level. Mulhern suggests that IQ drops 12−14 points in patients receiving RT.^[18]^ While a similar study by Rutkowski *et al*. in 2005, looking at the impact of chemotherapy on IQ, showed a decrease in general intelligence compared to healthy controls.^[20]^In our study, while duration since completion of adjuvant therapy did not have any significance in term of QoL, cognitive functioning or IQ score, it showed a trend towards significance in Movement and Balance dimension of QoL (child reported score; *P* = 0.083 and parent-reported score; *P* = 0.082), cognitive problem dimension in parent-reported scores (*P* = 0.053).

Various studies in literature looked at aspects like going back to school, type of school attended post-treatment, level, and extent of education, as well as aptitude of these groups of children. Bhat *et al*. studied, HRQOL of children with brain tumors in a cohort of 134 patients and reported that 70.1% of children went back to a normal school, while 29.9% required special education services^[17]^ Hoppe-Hirsch *et al*. reported that, at 5 years post-treatment, 55% of children were in special education, compared to 80% at 10 years.^[21]^ In our study, 61.70% went back to school following completion of treatment. If we assess the number of eligible children who went back to school, the percentage increases to 72.5% (29/40). Our data showed that the majority of the children in our cohort went back to school following therapy, though often with a delay of one academic year.

We analyzed maternal education separately, as our cohort consisted of children in the age group between 1-18 years and in the Indian scenario, the mother spends most of the time with her children during formative years. Very few studies have looked at specifically regarding the influence of parental education on QoL and IQ of children. Sato *et al*. reported that 39% of parents took school education, while 61.1% of parents took higher education in college or university and HRQOL is not affected by parental education (*P* = 0.17).^[22]^ In our study, the cognitive problem (*P* = 0.043) according to parent-reported scores and worry (*P* = 0.032) as per child-reported scores were significantly affected by the level of maternal education. Mothers with higher education in terms of college or above see lesser cognitive problems in their children.

## Quality of life assessment in children

Previous studies on HRQOL have smaller sample sizes or have used an instrument which either surveyed parent or patient only. With cognitive deficits and developmental delays that occur with adjuvant therapies for malignant tumors, surveying only parents or children may not give a complete overview of the problem. With PedsQL questionnaire, rapid and complete measurement of HRQOL was possible in both children and parents and also in children with reduced attention span. As PedsQL consists of various domains related to the daily life of children and their activities, it was a fruitful exercise to perform in order to understand impact of this disease process on their Quality of life. We noted that movement and balance were influenced by the recurrence, as expected. In the domain of worry, which evaluates the extent to which children and parents are concerned about the effectiveness of treatment, side effects, and outcomes in terms of cure as well as recurrence, we found that higher maternal education resulted in lesser worry, implying a better understanding of the disease process, encouraging children to have more freedom and restore confidence in them about the outcomes. Sato *et al*., reported that parents with high school graduation report lesser QoL in their children compared to college graduate parents, which is also our observation^[22]^ In parent-reported scores, higher LPS at discharge makes parents have lesser worry about the cure.

Studies evaluating QoL in PFBT in literature have so far specifically not examined the association between neurocognitive functioning and the impact of clinical parameters on it.When cognitive functioning scores were compared against IQ in our study, it is evident that the children with below-average IQ had lower cognitive function scores compared to those with higher IQ. Interestingly, even in the cohort of children with low IQ, the cognitive functioning scores were good enough for daily activities.

Maddrey *et al*. have reported that both survivors and caregivers do not report significant impairment in QoL scores. But when the actual neuropsychological assessment is done, these children perform poorly on measures of cognitive ability, attention, and memory which is reflected in poor overall IQ scores^[23]^

## Neurocognitive functions

There can be various factors influencing the current level of neurocognitive functioning in these children.It could be due to genetic factors, cognitive stimulation the child is receiving from the environment to side effects of treatment modalities like radiation. Hoppe-Hirsch *et al*., compared influence of RT and surgery on intellectual outcomes and showed that at 10 years follow up, 56% of children with ependymoma who received local RT had IQ more than 90, while only 10% of children of medulloblastoma had IQ above 90 at the same time.^[21]^As the majority of children in our study had medulloblastoma, it could be the reason for poor overall IQ scores of the cohort. Out of 37 cases of medulloblastoma in our study, 62.2% had below-average IQ and among 10 children with anaplastic ependymoma, 40% of children had below-average IQ. Nevertheless, histopathology did not have a significant impact on IQ outcomes in our study (*P* = 0.286).

We noted that children who had better IQ had a younger mean age at presentation. In contrary, various Western studies have noted, that younger age at onset of treatment leads to poor long-term IQ scores.^[18,24,25]^ A possible hypothesis is an improvement in target-specific RT can spare the normal surrounding brain from its impact and also neuroplasticity in a younger age which protects these children form a decline in IQ.

In our study, 84.61% of children coming from APL families were of average or above average IQ. This explains the importance of environment and psycho-social milieu that is available for these children from well to do a familial background. Kulkarni *et al*., noted that lower family income group has a negative impact on QoL of children, which is similar to our finding.^[16]^ However, Sato *et al*., on HRQOL in children with brain tumors, demonstrated that subjective opinion regarding the economic status and life does not influence parent’s perception of HRQoL.^[22]^

## Conclusions

This study noted that various clinical and social factors like LPS at discharge, maternal education, and family income influenced the QoL of children with malignant PFBT. Most of the children had higher scores than the median in cognitive functioning and were able to perform daily activities. However, they are not representative of actual neurocognitive tasks based on performance or IQ scores. These children would require intensive cognitive rehabilitation to improve their performance. An active and regular QoL and cognitive function assessment of children during regular follow-up, in addition to imaging, will enable us for modifying existing tumor-related treatment modalities and formulating newer ones for timely intervention in these groups of children for their rehabilitation and integration into society.

## Figures and Tables

**Table 1:** Clinical characteristics

Variable	Value
Age at presentation [Mean (Range)]	8.2 (1-18) years
Age at evaluation [Mean (Range)]	10.89 (3-21) years
Gender	
Male	31 (66%)
Female	16 (34%)
Location	
Midline	38 (80.9%)
Lateral (Hemispheric)	9 (19.2%)
Lansky score at presentation	
≤70	46 (97.9%)
>70	1 (2.1%)
Lansky score at discharge	
≤70	17 (36.2%)
>70	30 (63.8%)
Radiotherapy	
Yes	46 (97.9%)
No	1 (2.1%)
Chemotherapy	
Yes	31 (66%)
No	16 (34%)
Recurrence	
Yes	9 (19.1%)
No	38 (80.9%)
Pre-treatment Schooling	
Yes	29 (61.70%)
No	18 (38.29%)
Post-treatment Schooling	
Yes	29 (61.70%)
No	18 (38.29%)
Parental Education	
Father	44 (93.61%)
Mother	36 (76.59%)
Both	35 (74.46)

**Table 2:** Age group-wise QoL scores

Age	2-4 years Mean (SD)	5-7 years Mean (SD)		8-12 years Mean (SD)		13-18 years Mean (SD)	
QoLDimensions	Parent-reported scores	Child-reported scores	Parent-reported scores	Child-reported scores	Parent-reported scores	Child-reported scores	Parent-reported scores
Cognitive Problems	-	88.89 (18.003)	55.07 (13.65)	71.33 (16.21)	59.72 (23.19)	73.11 (23.18)	75.56 (26.05)
Pain and hurt	85 (13.69)	100 (0)	83.33 (25.97)	80.39 (16.65)	74.61 (18.38)	84.31 (18.37)	83.82 (16.79)
Movement and balance	63.33 (40.22)	91.67 (20.41)	71.87 (38.82)	74.02 (30.74)	69.61 (29.01)	65.68 (35.58)	66.66 (38.86)
Procedural anxiety	15 (22.36)	25 (27.39)	9.37 (12.94)	33.33 (23.57)	32.35 (22.02)	61.76 (30.77)	63.72 (38.52)
Nausea	69 (23.29)	70 (20.98)	58.75 (16.85)	74.70 (13.52)	61.98 (15.48)	76.08 (18.03)	79.70 (20.19)
Worry	55 (27.39)	85 (14.91)	42.71 (20.14)	68.14 (15.93)	57.84 (21.34)	72.55 (19.27)	66.17 (22.14)
Cognitive functioning scale	62.92 (16.16)	96 (8.94)	57.02 (15.97)	74.47 (16.39)	58.67 (22.25)	75.68 (21.53)	74.21 (26.90)

QoL: Quality of life

**Table 3:** Variables influencing cognitive problem

Clinical parameters	Child-reported scores		Parent-reported scores	
	Mean (SD)	*P*	Mean (SD)	*P*
Age groups				
2-4 years	-		-	
5-7 years	88.89 (± 18.00)	-	55.07 (± 13.65)	
8-12 years	71.33 (± 16.21)		59.72 (± 23.19)	
13-18 years	73.11 (± 23.19)		75.57 (± 26.05)	
Tumor location				
Brain stem	68.57 (± 27.36)		59.40 (± 14.80)	
Cerebellar	79.16 (± 10.20)	0.844	60.25 (± 12.49)	0.789
Vermian	74.86 (± 20.74)		67.99 (± 27.48)	
Lansky score at discharge				
≤70	69.30 (± 21.71)		48.56 (± 17.24)	
>70	77.64 (± 19.09)	0.257	75.01 (± 22.89)	0.001
Chemotherapy				
Yes	75.10 (± 18.85)		64.99 (± 24.36)	
No	74.20 (± 23.17)	0.989	67.19 (± 25.50)	0.808
Recurrence				
Yes	72.10 (± 18.45)		76.01 (± 30.22)	
No	75.27 (± 20.74)	0.705	63.58 (± 23.02)	0.306
Duration post-adjuvant therapy				
<12 months	72.16 (± 17.96)		55.36 (± 23.11)	
>12 months	75.95 (± 21.36)	0.492	70.76 (± 23.89)	0.053
Shunt				
Yes	75.42 (± 19.60)		64.15 (± 24.01)	
No	71.42 (± 24.06)	0.577	73.32 (± 27.04)	0.419
Socioeconomic status				
BPL	74.31 (± 19.68)		62.35 (± 24.18)	
APL	75.95 (± 22.65)	0.681	75.96 (± 23.54)	0.170
Maternal education				
No/Primary/Secondary	72.16 (± 19.34)	0.179	60.17 (± 22.05)	0.043
Degree	80.70 (± 21.66)		78.77 (± 25.75)	

APL: above poverty line; BPL: below poverty line

**Table 4:** Variables influencing movement and balance

Clinical parameters	Child-reported scores		Parent-reported scores	
	Mean (SD)	*P*	Mean (SD)	*P*
Age groups				
2-4 years	-		63.33 (± 40.22)	
5-7 years	91.66 (± 20.41)	-	71.87 (± 38.81)	-
8-12 years	74.02 (± 30.74)		69.60 (± 29.01)	
13-18 years	65.68 (± 35.59)		66.66 (± 38.86)	
Tumor location				
Brain stem	66.67 (± 47.14)		55.55 (± 43.99)	
Cerebellar	79.16 (± 24.58)	0.962	77.77 (± 25.68)	0.664
Vermian	72.98 (± 31.70)		67.96 (± 35.10)	
Lansky score at discharge				
≤70	63.09 (± 38.35)		56.86 (± 41.58)	
>70	78.52 (± 27.71)	0.202	74.27 (± 28.65)	0.198
Chemotherapy				
Yes	77.56 (± 28.26)		69.89 (± 32.53)	
No	64.88 (± 38.28)	0.323	65.10 (± 39.11)	0.825
Recurrence				
Yes	55.95 (± 17.15)		39.81 (± 32.48)	
No	76.77 (± 33.64)	0.036	75 (± 31.83)	0.007
Duration post adjuvant therapy				
<12 months	60.25 (± 36.03)		58.75 (± 36.72)	
>12 months	79.32 (± 28.90)	0.083	75.31 (± 31.73)	0.082
Shunt				
Yes	73.23 (± 33.38)		66.87 (± 33.85)	
No	72.61 (± 28.35)	0.760	75 (± 39.59)	0.347
Socioeconomic status				
BPL	69.17 (± 33.70)		62.99 (± 35.36)	
APL	85 (± 25.09)	0.165	82.05 (± 29.23)	0.070
Maternal education				
No/Primary/Secondary	72.32 (± 31.91)	0.704	63.05 (± 35.06)	0.142
Degree	75 (± 34.26)		77.45 (± 32.64)	

APL: above poverty line; BPL: below poverty line

**Table 5:** Variables influencing worry

Clinical parameters	Child-reported scores		Parent-reported scores	
	Mean (SD)	*P*	Mean (SD)	*P*
Age groups				
2-4 years	-		55 (± 27.39)	
5-7 years	85 (± 14.91)	-	42.71 (± 20.13)	-
8-12 years	68.13 (± 15.93)		57.84 (± 21.34)	
13-18 years	72.54 (± 19.27)		66.17 (± 22.14)	
Tumor location				
Brain stem	71.67 (± 20.92)		48.61 (± 13.34)	
Cerebellar	58.33 (± 11.78)	0.137	62.03 (± 25.03)	0.505
Vermian	74.71 (± 17.46)		58.59 (± 23.71)	
Lansky score at discharge				
≤70	66.02 (± 16.82)		43.62 (± 16.01)	
>70	75.31 (± 17.71)	0.156	66.11 (± 22.31)	0.002
Chemotherapy				
Yes	74 (± 16.55)		59.67 (± 24.73)	
No	69.04 (± 20)	0.309	54.68 (± 18.99)	0.402
Recurrence				
Yes	65.47 (± 16.26)		53.70 (± 32.30)	
No	73.69 (± 17.97)	0.246	58.99 (± 20.45)	0.833
Duration post-adjuvant therapy				
<12 months	70.51 (± 16.53)		52.5 (± 22.63)	
>12 months	73.07 (± 18.60)	0.784	62.03 (± 22.57)	0.153
Shunt				
Yes	73.17 (± 16.90)		55.55 (± 21.48)	
No	67.85 (± 22.27)	0.389	69.79 (± 27.07)	0.146
Socioeconomic status				
BPL	69.72 (± 16.74)		55.39 (± 22.37)	
APL	80.55 (± 19.54)	0.105	64.74 (± 23.60)	0.213
Maternal education				
No/Primary/Secondary	68.45 (± 16.25)	0.032	54.16 (± 21.30)	0.105
Degree	81.81 (± 18.57)		64.70 (± 24.57)	

APL: above poverty line; BPL: below poverty line

**Table 6:** Variables influencing cognitive functioning

Clinical parameters	Child-reported scores		Parent-reported scores	
	Mean (SD)	*P*	Mean (SD)	*P*
Age groups				
2-4 years	-		62.91 (± 16.16)	
5-7 years	96 (± 8.94)	-	57. 02 (± 15.97)	-
8-12 years	74.47 (± 16.39)		58.67 (± 22.25)	
13-18 years	75.68 (± 21.53)		74.21 (± 26.90)	
Tumor location				
Brain stem	72.91 (± 27.53)		59.99 (± 16.30)	
Cerebellar	78.47 (± 10.68)	0.909	64.16 (± 10.77)	0.888
Vermian	78.27 (± 19.82)		65.48 (± 26.96)	
Lansky score at discharge				
≤70	75.12 (± 20.93)		48.82 (± 16.90)	≤0.001
>70	79.23 (± 18.38)	0.498	73.89 (± 21.83)	
Chemotherapy				
Yes	75.83 (± 19.59)		63.26 (± 23.93)	
No	78.83 (± 19.24)	0.616	67.18 (± 22.92)	0.669
Recurrence				
Yes	73.21 (± 14.99)		67.12 (± 30.65)	
No	78.75 (± 20.03)	0.484	64.01 (± 21.78)	0.813
Duration post-adjuvant therapy				
<12 months	77.77 (± 16.29)		57.85 (± 19.88)	
>12 months	77.75 (± 20.76)	0.776	69.84 (± 24.92)	0.085
Shunt				
Yes	78.75 (± 18.81)		63.28 (± 22.43)	
No	73.21 (± 21.63)	0.580	70.99 (± 28.37)	0.399
Socioeconomic status				
BPL	77.13 (± 19.77)		61.39 (± 23.06)	
APL	79.58 (± 18.15)	0.796	72.85 (± 23.11)	0.145
Maternal education				
No/Primary/Secondary	74.75 (± 19.49)	0.150	59.73 (± 22.01)	0.087
Degree	84.52 (± 17.27)		72.96 (± 24)	

APL: above poverty line; BPL: below poverty line

## Key Message

Children with malignant posterior fossa tumors had diverse deficits in QoL and neuropsychological assessment at follow up. QoL scores were above median values in most parameters. A higher Lansky performance score and maternal education were associated with lesser cognitive problems in children. 57.5% children had below average IQ in this cohort; however the children had better QoL scores of more than 50 in the cognitive functioning scale. A majority of children went back to school following therapy with a delay of one academic year.
